# Supplementary material for: Bamboo-like dual-phase nanostructured copper composite strengthened by amorphous boron framework
Source: Nat Commun. 2023 Aug 10;14:4836. doi: 10.1038/s41467-023-40580-8 (PMC10415290; doi:10.1038/s41467-023-40580-8)
Supplement: Supplementary file 1 — Supplementary Information. [file 41467_2023_40580_MOESM1_ESM.pdf]

Supplementary Information for
Bamboo-like dual-phase nanostructured copper composite strengthened by amorphous boron framework

Hang Lv[1]†, Xinxin Gao[1]†, Kan Zhang[1]*, Mao Wen[1], Xingjia He[1], Zhongzhen Wu[2], Chang Liu[3]*, Changfeng Chen[4], Weitao Zheng[1]

[1]State Key Laboratory of Superhard Materials, Department of Materials Science and Key Laboratory of Automobile Materials, MOE, Jilin University, Changchun 130012, China.

[2]School of Advanced Materials, Peking University Shenzhen Graduate School, Shenzhen, 518055, China.

[3]International Center for Computational Methods and Software, College of Physics, Jilin University, Changchun 130012, China.

[4]Department of Physics and Astronomy, University of Nevada, Las Vegas, Nevada 89154, USA.

†These authors contributed equally to this work.

**Corresponding author. Email: kanzhang@jlu.edu.cn; liuchang127@jlu.edu.cn*

Table of content

I. Supplementary Discussion

II. Supplementary Table

III. Supplementary Figures

Supplementary Figure 1. XPS spectra of Cu-B films with varying B concentration.

Supplementary Figure 2. XRD patterns of pure Cu, pure B, and Cu-B films.

Supplementary Figure 3. Representative TEM images of structural overview of the Cu-26.5 at.% B film.

Supplementary Figure 4. Statistical data analysis of grain size and grain spacing from TEM images of the plan-view of the Cu-26.5 at.% B film.

Supplementary Figure 5. Lattice orientation analysis of representative HRTEM images of a columnar Cu grain of the Cu-26.5 at.% B film.

Supplementary Figure 6. EELS results for the Cu-26.5 at.% B film in cross-sectional view.

Supplementary Figure 7. Solid solution formation energy calculations and AIMD simulations.

Supplementary Figure 8. TEM analysis of the Cu-10.3 at.% B film's cross-sectional structures.

Supplementary Figure 9. TEM analysis of the Cu-36.1 at.% B film's cross-sectional structures.

Supplementary Figure 10. TEM analysis of the Cu/B multilayer film's cross-sectional structures

Supplementary Figure 11. Representative AFM images of the indentation site of the "bamboo-like" dual-phase Cu-B nanocomposite film.

Supplementary Figure 12. Representative DF-STEM images of the cross-sectional view of the indentation site of the "bamboo-like" dual-phase Cu-B nanocomposite film.

Supplementary Figure 13. TEM analysis of the indented Cu/B multilayer film's cross-sectional structures.
Supplementary Figure 14. Phonon dispersion curves.
IV. Supplementary References

I. Supplementary Discussion

Analysis of the classic boundary strengthening effect

The morphology near the indentation site is known to affect the hardness to strength ratio [1]. When a pile-up behavior occurs (Fig. 3 and Fig. S6), hardness (H) is approximately 3 times the strength (σ): $H \approx 3\sigma$. The strength of a nanocrystalline metal can be divided into two components, *i.e.*, the thermal component (σ_{th}) and the athermal component (σ_{ath}) [2]: $\sigma = \sigma_{th} + \sigma_{ath}$. The term σ_{th} includes lattice friction ($\sigma_0 \approx 116$ MPa) and solid solution strengthening (σ_{ss}). Since boron is hardly dissolved into the Cu lattice (Fig. 1 and Fig. S3), $\sigma_{ss} \approx 0$. The term σ_{ath} stems from long-range defects such as dislocations (σ_{dis}) and boundaries (σ_B). Due to the low initial dislocation density inside the copper grains, σ_{dis} can be ignored. The term σ_B is described by the GBs (σ_{GB}) and segregation atoms because of the GB segregation effect [3]:

$$\sigma_B = \frac{\sigma_{GB}}{(1-t)+kE_{GB}\sigma_{GB}t} \cdots\cdots (1)$$

where k is a fitting parameter, E_{GB} is the specific GB energy (0.041 J/mol), and $t=N_{GB}/N_{total}$, where N_{GB} and N_{total} are the number of atoms at the GBs and the total number of atoms, respectively. Since our grain size (d) is larger than the critical grain size (10 nm) for the Hall-Petch effect, σ_{GB} can be determined by [4]:

$$\sigma_{GB} = \sigma_0 + k_{GB} d^{-\frac{1}{2}} \cdots\cdots (2)$$

where $k_{GB} = 3478$ MPa nm$^{1/2}$. Taken together, the hardness of the nanocolumnar copper based on the classic boundary strengthening analysis is $H \approx 3(\sigma_0 + \sigma_{ss} + \sigma_{dis} + \sigma_B) = 4.698$ GPa.

Analysis of factors influencing the strain rate sensitivity (SRS) index *m*

The grain size [5] and microstructure [6] are the key contributing factors in setting the strain rate sensitivity (SRS) index *m*. As reported by Chen *et al.* [5], when grain size is reduced to nanometer scale, a large number of GBs and/or sub-grain boundaries can serve as significant obstacles to dislocation motion, resulting in an increase of *m*. However, the *m* value in this work exhibits a contrasting decreasing trend with decreasing grain size (Fig. 4). Therefore, the main factor determining *m* here is attributed to the microstructure of amorphous boron framework TGBs in the "bamboo-like" dual-phase nanocomposite Cu-B film rather than the grain size.

II. Supplementary Table

Supplementary Table 1. Experimental parameters (input power of Cu/B target), composition measured by XPS, residual stress, grain size calculated by XRD, hardness and modulus of the pure Cu and Cu-B alloy films with variable B concentrations.

Cu/B	Composition			Grain size	Residual stress	Modulus
(W)	Cu (at.%)	B (at.%)	B (wt.%)	(nm)	(GPa)	(GPa)
20/0	100	0	0	18.9	0.75	103.1 ± 1.6
20/150	89.7	10.3	1.9	8.1	-0.82	143.2 ± 1.7
20/250	73.5	26.5	5.8	9.5	-0.72	176.6 ± 4.6
20/350	63.9	36.1	8.8	6.5	-0.07	156.9 ± 3.3

We synthesized pure Cu and Cu-B films with varying B concentrations. It is achieved by manipulating the B target power across a range of values, namely 0, 150, 250, and 350 W. The resulting B concentrations are measured by XPS to be 0, 10.3, 26.5, and 36.1 at.%, which can be alternatively expressed as 0, 1.9, 5.8, and 8.8 wt.%, respectively. The grain size of the above films is calculated from the XRD results. The addition of element B causes a significant decrease in the grain size, from 18.9 nm for the pure Cu film to 8.1 GPa for the Cu-10.3 at.% B film. The grain size of Cu-B films does not show a regular pattern with increasing B concentration and is close to the critical grain size for Hall-Petch effect failure. The residual stresses in all films are small and do not exhibit a significant trend. The modulus of the pure Cu film is 103.1 ± 1.6 GPa, and increases to 143.2 ± 1.7 GPa and 176.6 ± 4.6 GPa when B concentrations are 10.3 at.% and 26.5 at.%, respectively.

III. Supplementary Figures

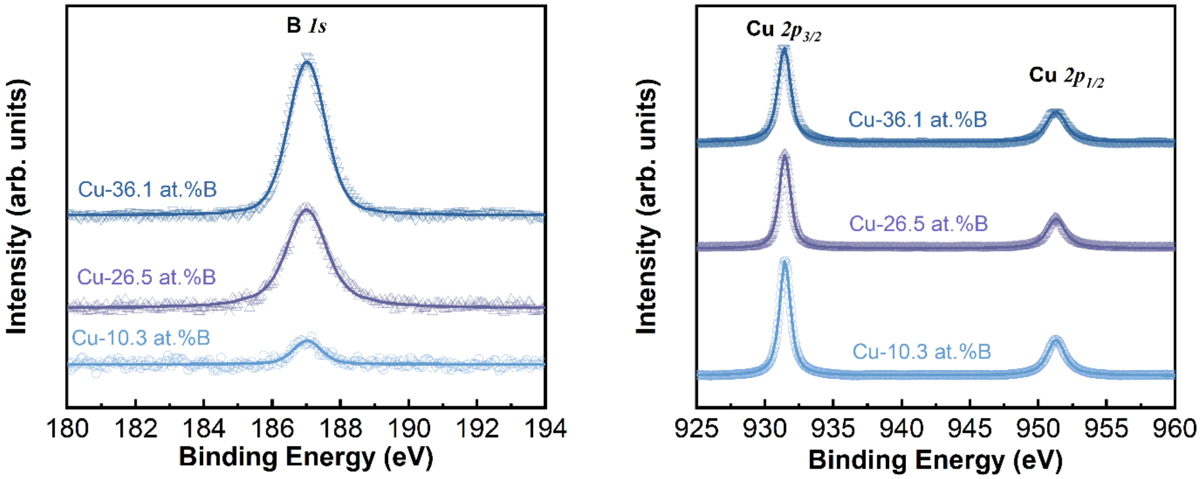

Supplementary Figure 1. XPS spectra of Cu-B films with varying B concentration. The B *1s* and Cu *2p* orbitals exhibit distinct peaks corresponding to B-B and Cu-Cu bonds, respectively. The concentration of B element in the films is determined by analyzing the peak areas.

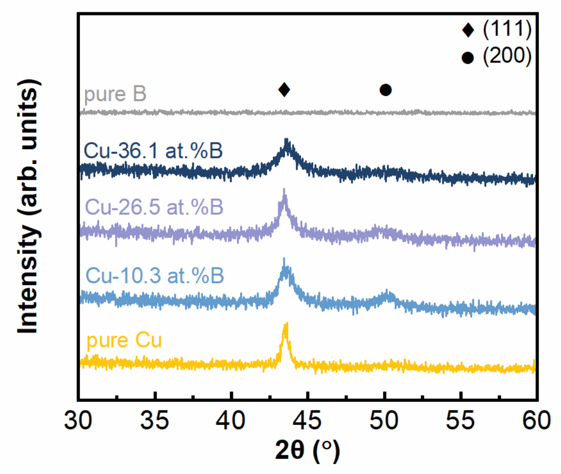

Supplementary Figure 2. XRD patterns of pure Cu, pure B, and Cu-B films. The pure Cu film shows a single fcc (111) peak, while the Cu-B films exhibit an additional fcc (200) peak. The intensity of the peak becomes progressively weaker with increasing B concentration. The pure B film demonstrates an amorphous structure. Grain size is calculated from the Cu(111) peak in the XRD graph by Scherer's formula [7]: $d = k\lambda/\beta cos\theta$, where λ is the wavelength of the X-ray, β is the full width at half maximum (FWHM) of the peak, θ is diffraction angle, and k is a constant.

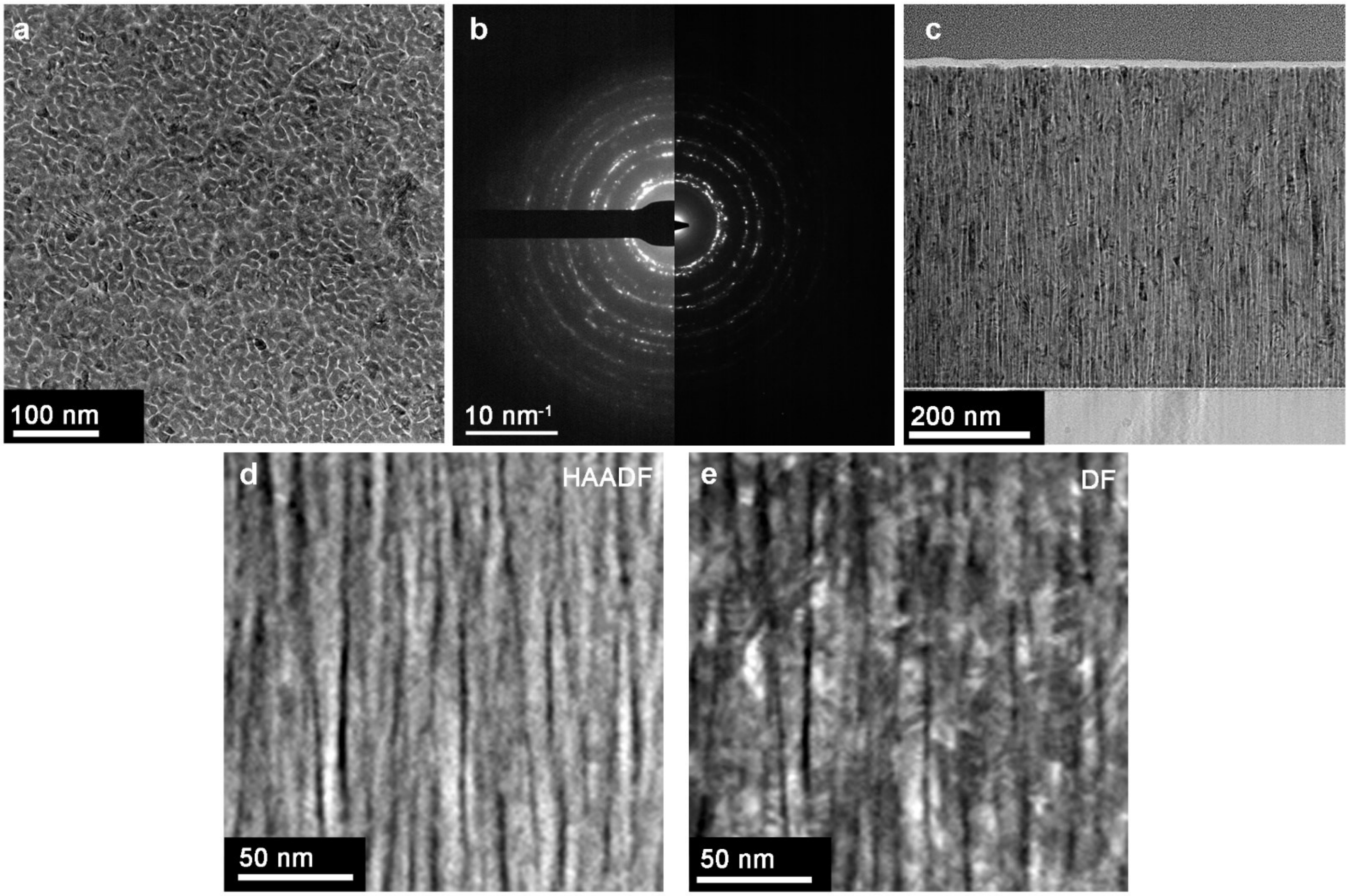

Supplementary Figure 3. Representative TEM images of structural overview of the Cu-26.5 at.% B film. (**a**) A representative TEM image of the plan-view showing a typical core-shell structure of the nanocrystal-amorphous composite. (**b**) A representative SAED image of the plan-view (left) and the cross-sectional view (right). The positions of the diffraction rings in the two images are exactly the same and conform to the standard polycrystalline fcc Cu. (**c**) A representative TEM image of the cross-sectional view. It shows an obvious columnar growth morphology. (**d**) A representative HAADF-STEM image of the cross-sectional view. The Z-contrast in the HAADF image is highly dependent on atomic number, thus distinguishing different elements. Since there are only two elements Cu and B in the films, the low contrast is B and the high contrast is Cu. (**e**) A DF-STEM image in the same region shown in (d). The contrast in DF image depends on the degree of conformity to Bragg relationship, and the position with better crystallinity has higher contrast. Compared with the corresponding position in (d), it is seen that the corresponding position of boron has poor crystallinity, indicating its amorphous nature.

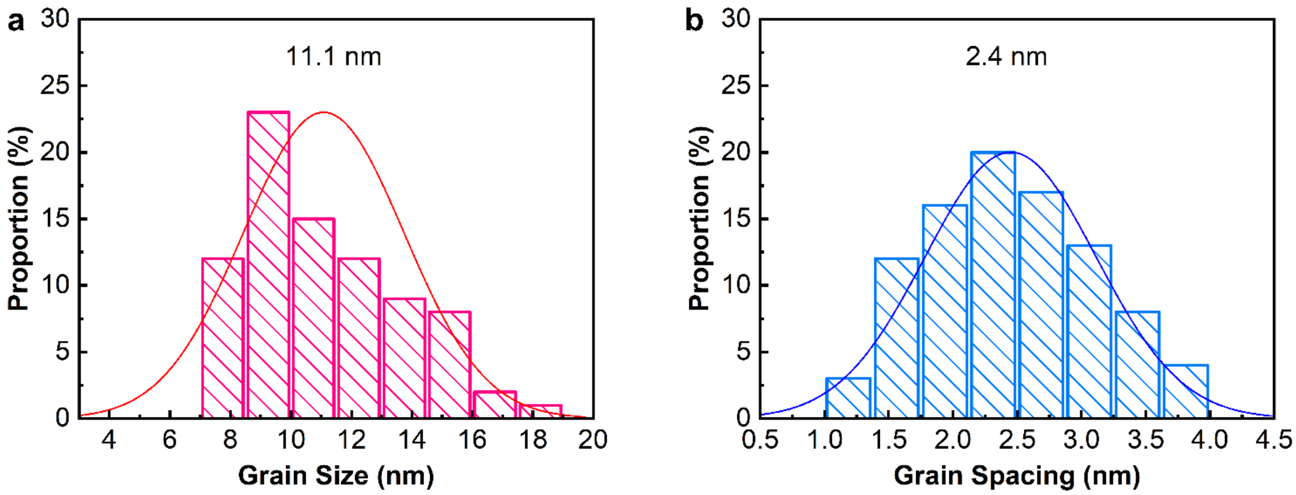

Supplementary Figure 4. Statistical data analysis of grain size and grain spacing from TEM images of the plan-view of the Cu-26.5 at.% B film. (**a**) Distribution of grain sizes. The average grain size is 11.1 nm, which is within the critical grain size range for the Hall-Petch effect of Cu. (**b**) Distribution of grain spacings. The average grain spacing is 2.4 nm, representing the thickness of the TGBs. At least 100 grains were examined.

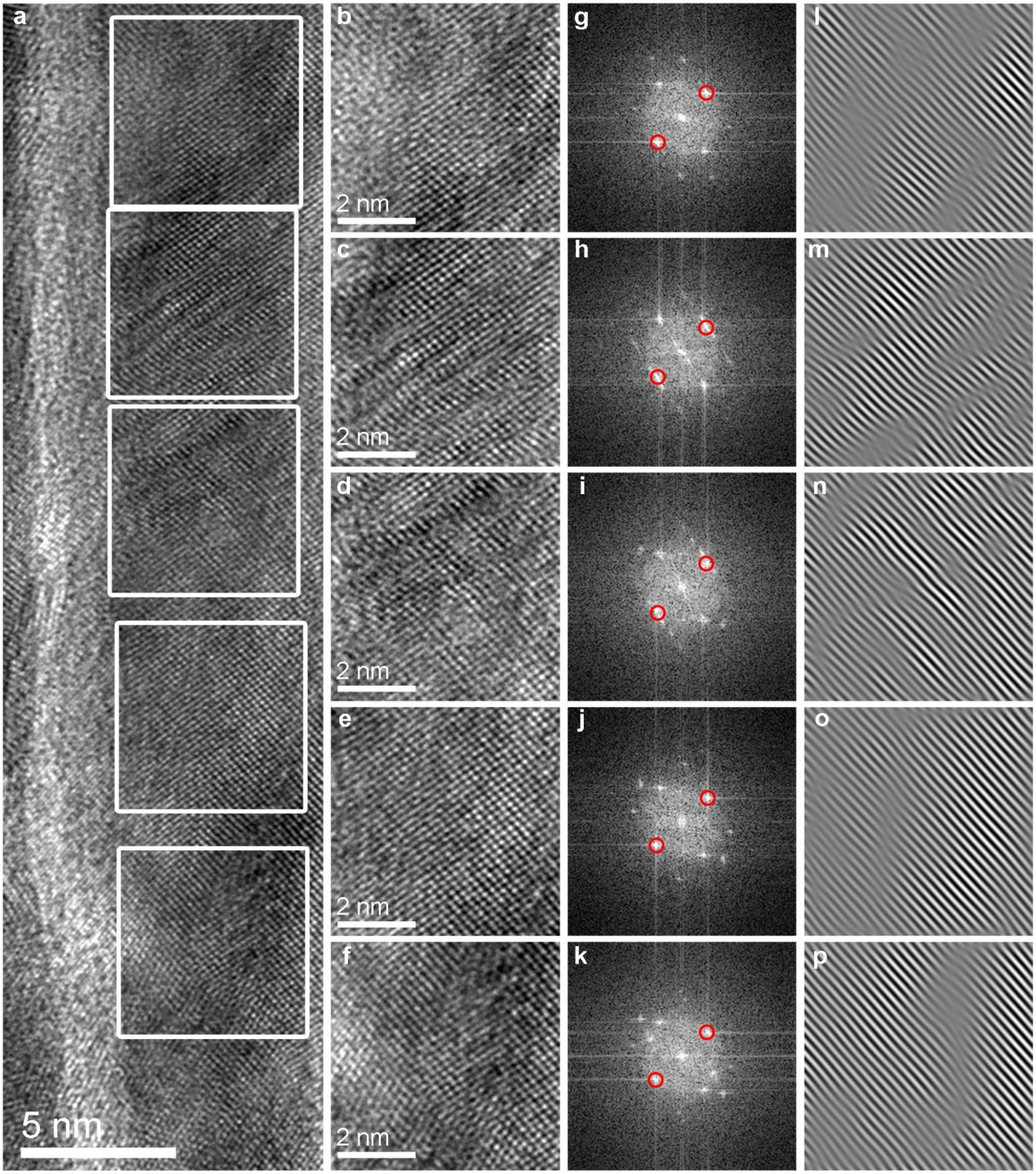

Supplementary Figure 5. Lattice orientation analysis of representative HRTEM images of a columnar Cu grain of the Cu-26.5 at.% B film. (**a**) The HRETM image of the grain. A grain and adjacent amorphous grain boundaries are shown. (**b~f**) Enlarged HRTEM images of the top to bottom region inside the grain. (**g~k**) The FFT results of the HRTEM images on left. The red circles represent the diffraction spots on the (111) plane. (**l~p**) The IFFT results after filtering the (111) spots of the FFT images on left. All (111) lattice fringes have exactly the same direction, showing high structural integrity of the columnar grain, and only few dislocations are observed.

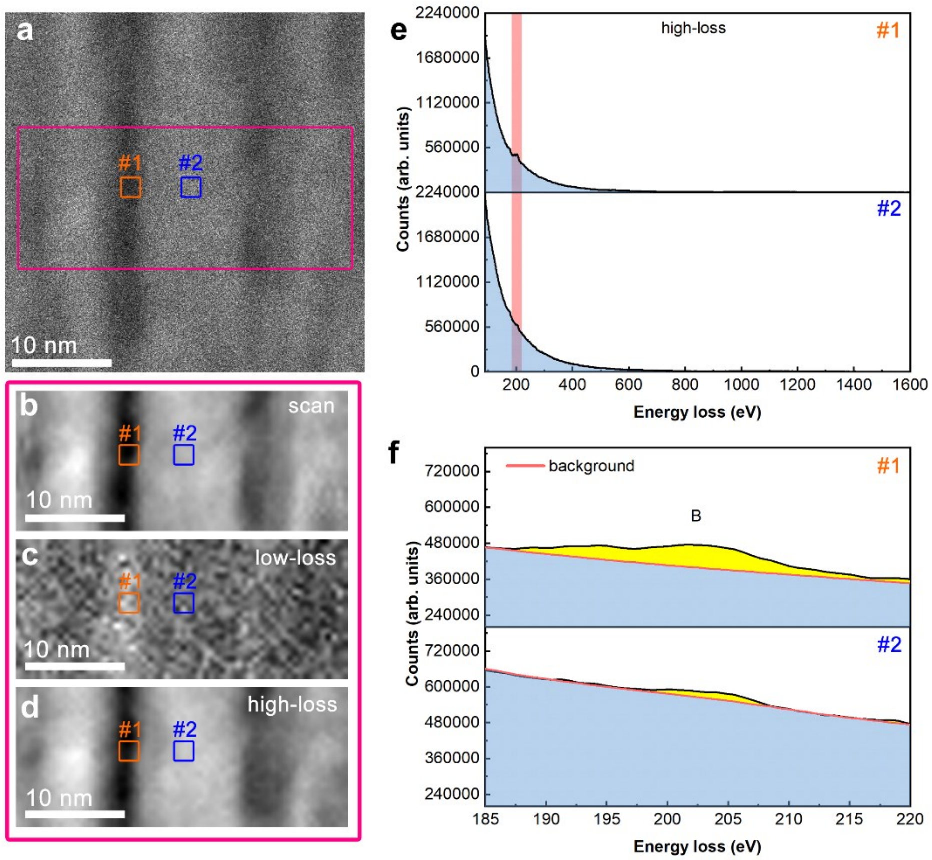

Supplementary Figure 6. EELS for the "bamboo-like" dual-phase Cu-B nanocomposite film in cross-sectional view. (**a**) The EELS imaging reveals discernible columnar arrangements within the film. (**b**, **c** and **d**) Scanned image, low-loss image and high-loss image, respectively, of the selected locations in A. Point scanning is performed at position #1 within the TGB and at position #2 within the grain. (**e**) High-loss partial spectra at positions #1 and #2, where a more significant peak at around 200 eV is clearly observed at position #1 compared to position #2, which can be identified as the peak of element B[8]. (**f**) A magnified image of the energy loss around 200 eV, showing that the peak area at position #1 is ~6.2 times larger than that at position #2 after subtracting the background. This result demonstrates the enrichment of element B at the TGB.

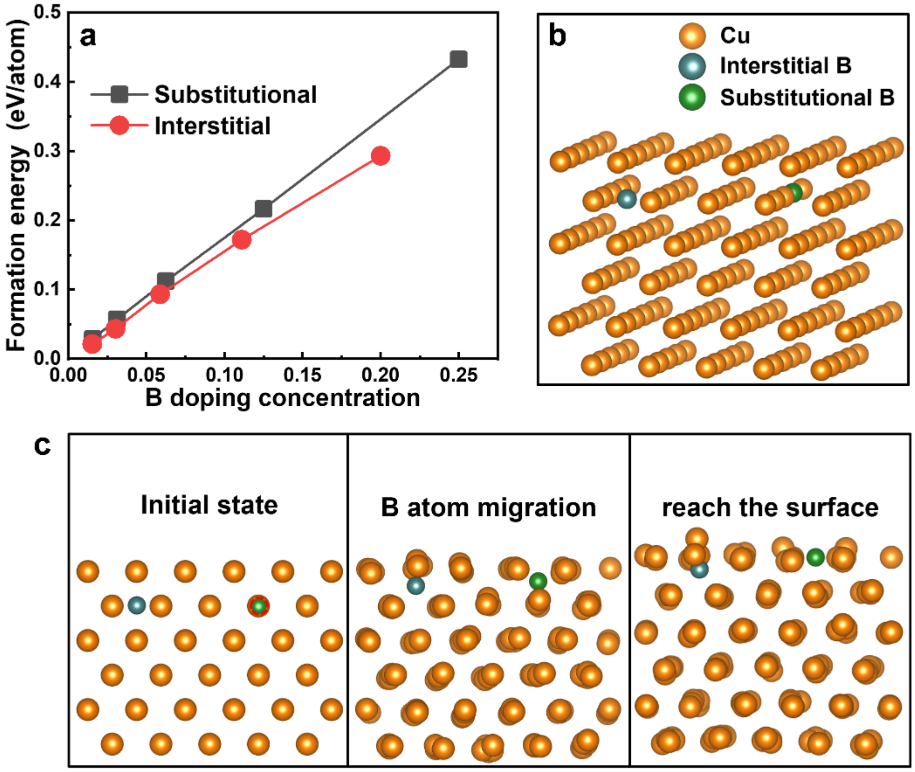

Supplementary Figure 7. Solid solution formation energy calculations and AIMD simulations. (a) The formation energy of Cu-B substitutional and interstitial solid solution as a function of B atom doping concentration. **(b)** 3D structure for AIMD simulations, including six layers with a total of 149 Cu atoms, one interstitial B atom, and one displacement B atom. **(c)** AIMD simulations of the movement of doped B atoms toward the surface at 400 K.

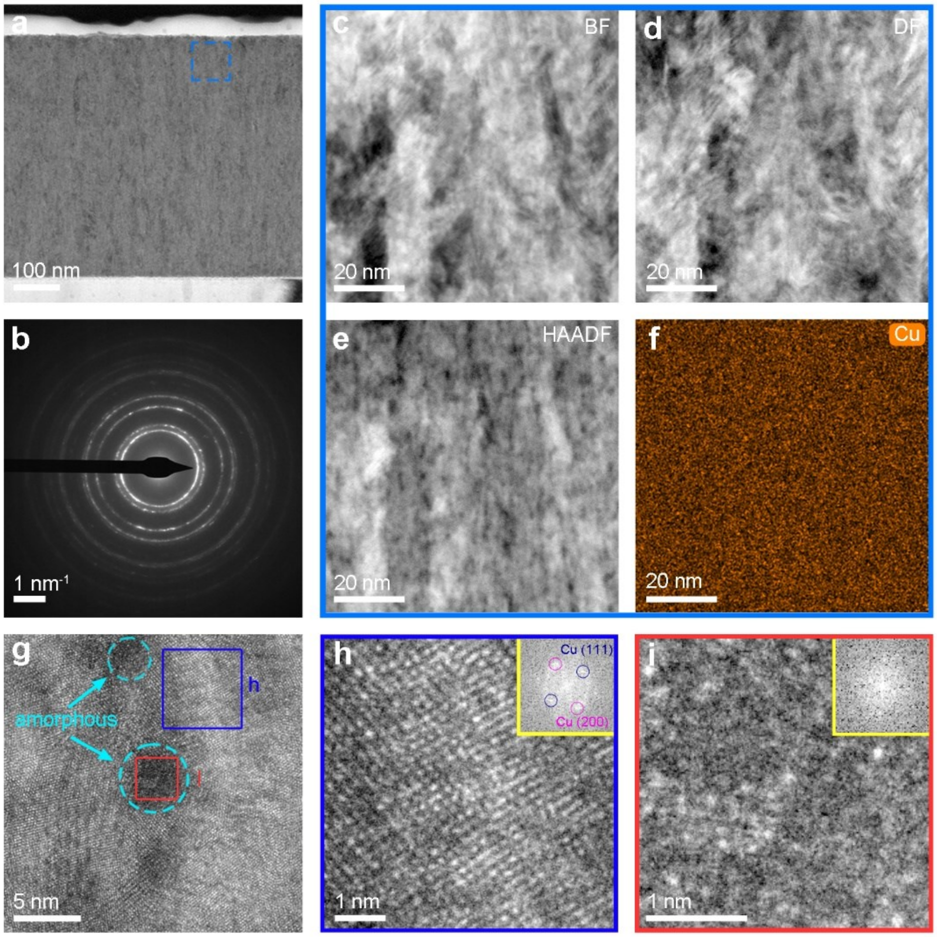

Supplementary Figure 8. TEM analysis of the Cu-10.3 at.% B film's cross-sectional structures. (**a**) The representative TEM image shows a morphology without significant features. (**b**) The SAED image shows a standard fcc polycrystalline diffraction ring. (**c** to **f**) The BF-STEM, DF-STEM, and HAADF-STEM images, along with Cu element mapping (shown at the same position), respectively, corresponding to the blue boxes in (a). These images reveal a featureless morphology with uniform distribution of Cu elements. (**g**) The representative HRTEM image shows slight amorphous phase distributed diffusely between Cu grains. (**h** and **i**) The HRTEM images of the crystalline and amorphous regions, respectively. The FFT images of the corresponding regions are shown in the upper right corner. The crystalline region shows a complete lattice stripe with FFT results containing Cu(111) and Cu(200) diffraction spots, indicating the standard fcc structure. The amorphous region shows disordered structure.

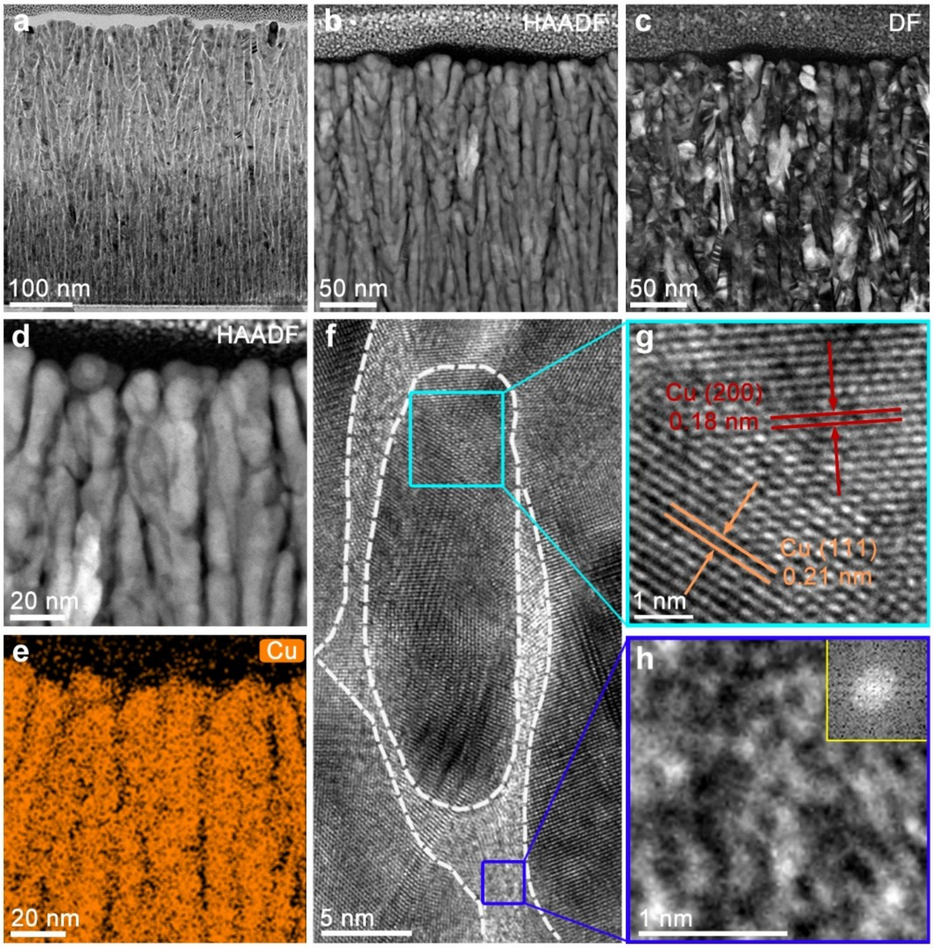

Supplementary Figure 9. TEM analysis of the Cu-36.1 at.% B film's cross-sectional structures. (**a**) The representative TEM image shows that the film exhibits a short columnar structure. (**b** and **c**) The representative HAADF-STEM and DF-STEM images show the nanograins embedded in the amorphous phase. (**d** and **e**) The HAADF-STEM image and the corresponding elemental mapping of Cu illustrate that the Cu element is mainly distributed inside the grains. (**f**) The representative HRTEM image presents the morphology of the single grain and surrounding area. (**g** and **h**) The HRTEM images of the crystalline and the amorphous regions, respectively. The lattice stripes of both Cu(111) and Cu(200) orientations are present inside the crystalline region. The FFT image of the amorphous region is shown in the upper right corner, presenting a distinct amorphous halo.

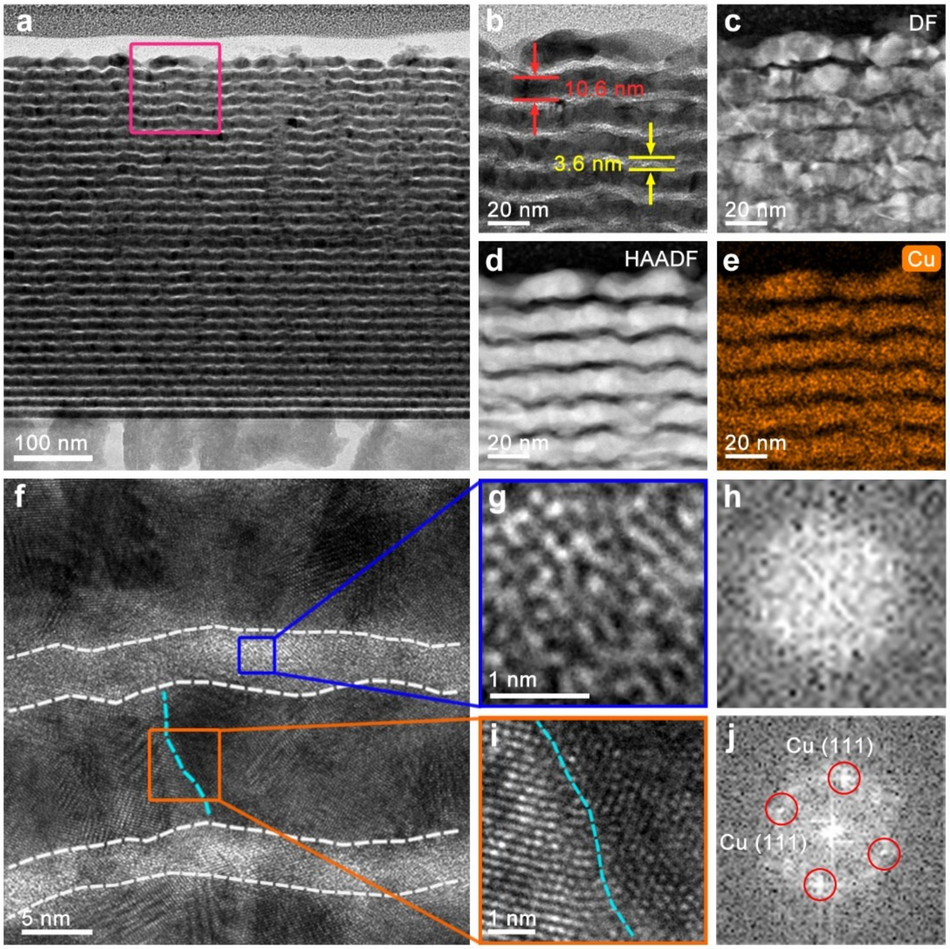

Supplementary Figure 10. TEM analysis of the Cu/B multilayer film's cross-sectional structures. (**a**) The representative TEM image shows a distinct layer structure. (**b** to **e**) The BF, DF-STEM, HAADF-STEM images, and the element mapping of Cu at the same position, respectively. The thickness of Cu layer and B layer is 10.6 nm and 3.6 nm, respectively. The elemental composition and structural dimensions are similar to those of the "bamboo-like" Cu-B film. (**f**) The representative HRTEM image containing both Cu layer and B layer. (**g**) HRTEM image of the B layer shows the amorphous structure. (**h**) The FFT image of (G) exhibits an amorphous halo. (**i**) HRTEM image of the Cu layer shows significant GBs. (**j**) The FFT image of (I) displays two sets of Cu (111) diffraction spots, indicating the presence of GBs.

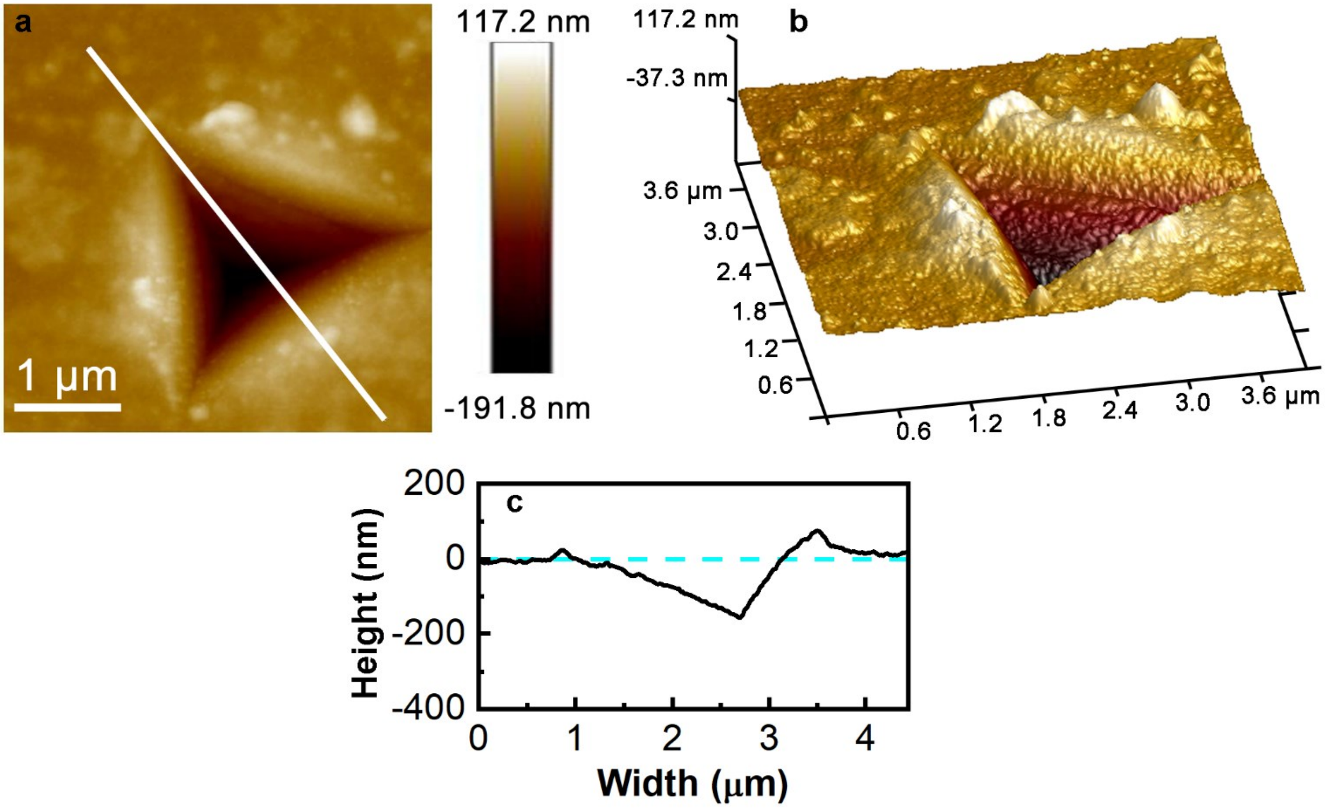

Supplementary Figure 11. Representative AFM images of the indentation site of the "bamboo-like" dual-phase Cu-B nanocomposite film. (**a**) AFM image of the indentation site. The white line passes through a vertex and is perpendicular to an edge. (**b**) 3D AFM image of the indentation site, revealing a pile-up behavior. (**c**) The height changes along the white line in (a). The pile-up height and indentation depth can be measured directly, which are consistent with the cross-sectional morphology of the indentation site.

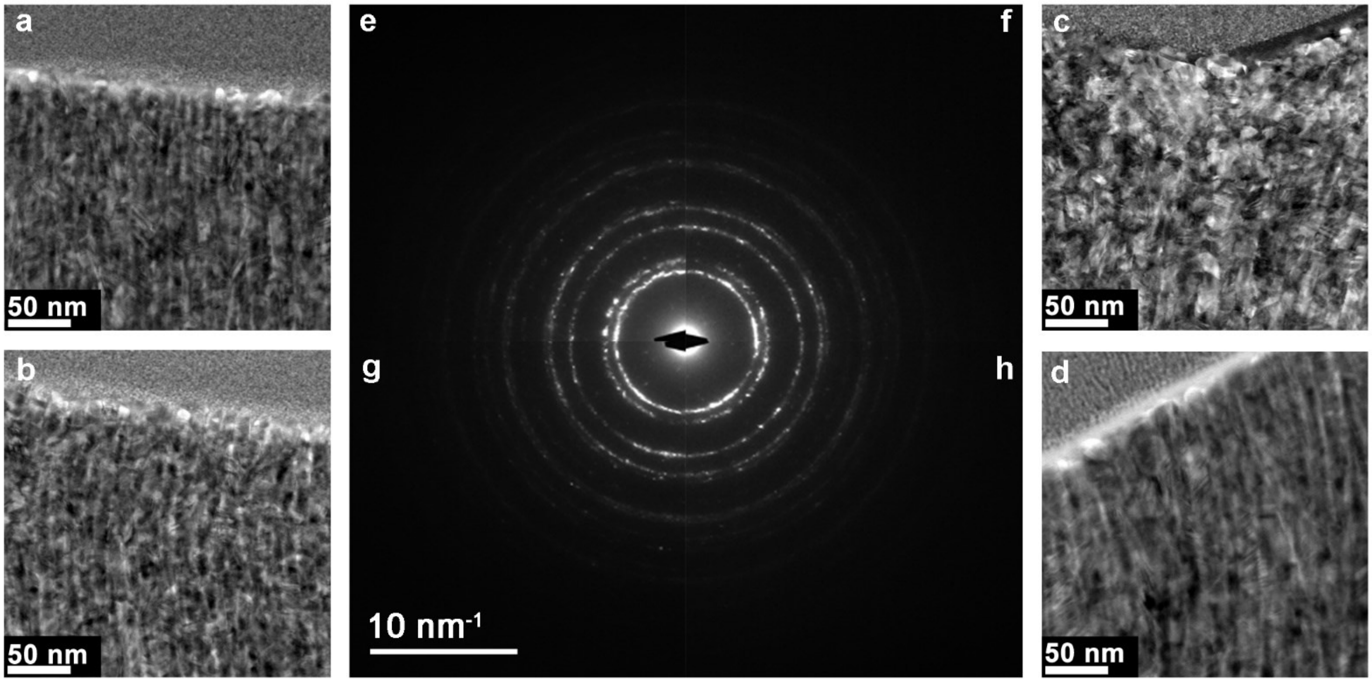

Supplementary Figure 12. Representative DF-STEM images of the cross-sectional view of the indentation site of the "bamboo-like" dual-phase Cu-B nanocomposite film. (**a~d**) DF-STEM images of different regions close to the surface under indentation. (**e~h**) SAED images of the corresponding positions shown in (a~d). The positions of the diffraction rings are exactly the same and conform to the standard polycrystalline fcc Cu.

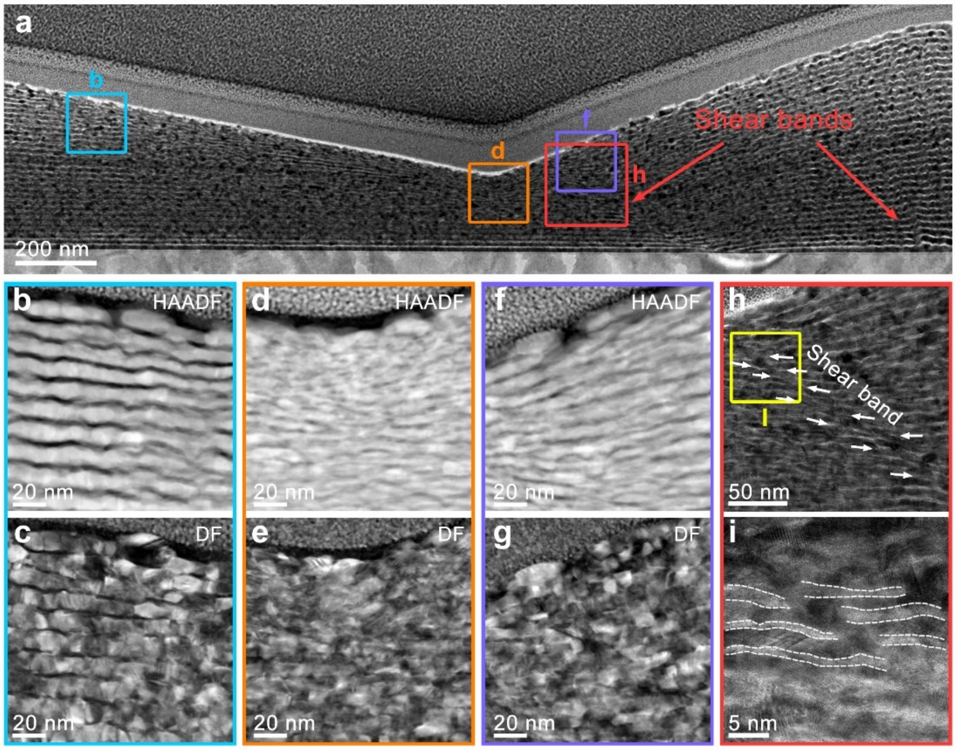

Supplementary Figure 13. TEM analysis of the indented Cu/B multilayer film's cross-sectional structures. (**a**) The representative TEM image. (**b** and **c**) HAADF-STEM and DF-STEM images of the region with lower plastic strains. (**d** and **e**) HAADF-STEM and DF-STEM images of the region with higher plastic strains. (**f** and **g**) HAADF-STEM and DF-STEM images of the region with the largest observed plastic strains. (**h**) TEM image of the shear band region. (**i**) Magnified HRTEM image of the yellow box in (h).

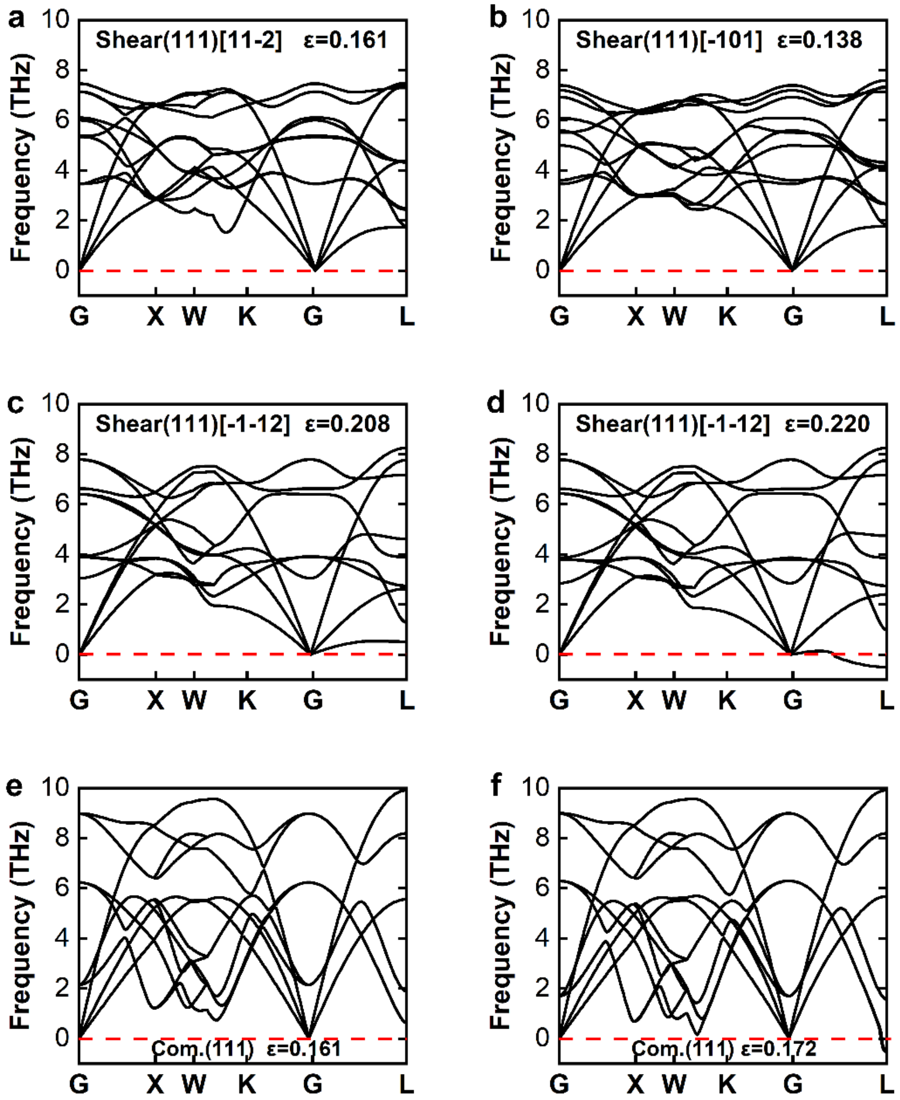

Supplementary Figure 14. Phonon dispersion curves. Calculated phonon dispersion curves for the strained FCC Cu **(a)** at the peak stress point under the (111)[11-2] Shear strain, **(b)** at the peak stress point under the (111)[-101] Shear strain, **(c, d)** under the (111)[-1-12] Shear strains, **(e, f)** under the [111] compressive strains. The appearance of imaginary phonon modes before reaching the peak stress in the latter two cases indicates early onsets of dynamic instability of the deformed structure. Our results show that the structures at the indicated peak stresses are dynamically stable under the (111)[11-2] and (111)[-101] shear strains, while under the (111)[-1-12] shear strains and [111] compressive strains, the maximum strains for dynamic stability are 0.208 and 0.161, respectively.

IV. Supplementary References

1. Zhang P, Li SX, Zhang ZF. General relationship between strength and hardness. *Mater Sci Eng, A* **529**, 62-73 (2011).
2. Li XG*, et al.* Tuning the microstructure and mechanical properties of magnetron sputtered Cu-Cr thin films: The optimal Cr addition. *Acta Mater* **151**, 87-99 (2018).
3. Vo NQ, Averback RS, Bellon P, Caro A. Limits of hardness at the nanoscale: Molecular dynamics simulations. *Phys Rev B* **78**, 241402-241406 (2008).
4. Chen XH, Lu L, Lu K. Grain size dependence of tensile properties in ultrafine-grained Cu with nanoscale twins. *Scr Mater* **64**, 311-314 (2011).
5. Chen J, Lu L, Lu K. Hardness and strain rate sensitivity of nanocrystalline Cu. *Scr Mater* **54**, 1913-1918 (2006).
6. Picu RC, Vincze G, Gracio JJ, Barlat F. Effect of solute distribution on the strain rate sensitivity of solid solutions. *Scr Mater* **54**, 71-75 (2006).
7. Holzwarth U, Gibson N. The Scherrer equation versus the 'Debye-Scherrer equation'. *Nature Nanotechnology* **6**, 534 (2011).
8. Lu Y-G, Turner S, Ekimov EA, Verbeeck J, Van Tendeloo G. Boron-rich inclusions and boron distribution in HPHT polycrystalline superconducting diamond. *Carbon* **86**, 156-162 (2015).
