# Supplementary material for: Bamboo-like dual-phase nanostructured copper composite strengthened by amorphous boron framework
Source: Nat Commun. 2023 Aug 10;14:4836. doi: 10.1038/s41467-023-40580-8 (PMC10415290; doi:10.1038/s41467-023-40580-8)
Supplement: Supplementary file 3 — Description of Additional Supplementary Files [file 41467_2023_40580_MOESM3_ESM.pdf]

Description of Additional Supplementary Files

File Name: Supplementary Movie 1
Description: In situ SEM pillar compression test of the “bamboo-like” dual-phase Cu-B nanocomposite film
